# Evaluation and Implementation of ListeningTime: A Web-Based Preparatory Communication Tool for Elderly Patients With Cancer and Their Health Care Providers

**DOI:** 10.2196/11556

**Published:** 2019-01-30

**Authors:** Janneke Noordman, Jeanine A Driesenaar, Inge R van Bruinessen, Johanneke EA Portielje, Sandra van Dulmen

**Affiliations:** 1 Nivel: Netherlands Institute for Health Services Research Utrecht Netherlands; 2 Department of Primary and Community Care Radboud University Medical Center Radboud Institute for Health Sciences Nijmegen Netherlands; 3 Department of Medical Oncology Leiden University Medical Center Leiden Netherlands; 4 Department of Oncology Hagaziekenhuis Den Haag Netherlands; 5 Faculty of Health and Social Sciences University of South-Eastern Norway Drammen Norway

**Keywords:** audio-facility, cancer patients, communication, internet, health care providers, videos, Web-based tool

## Abstract

**Background:**

Effective patient-provider communication is an important condition to deliver optimal care and it supports patients in coping with their disease. The complex and emotionally loaded setting of oncology care challenges both health care providers (HCPs) and patients in reaching effective communication. ListeningTime is developed for elderly patients with cancer and their oncological HCPs to help them (better) prepare the clinical encounter and overcome communication barriers. ListeningTime is a Web-based preparatory communication tool including modeling videos and has an audio-facility to listen back to recorded encounters.

**Objective:**

This study aims to evaluate the usability, perceived usefulness, and actual use of ListeningTime, through the eyes of elderly patients with cancer and their oncological HCPs. If highly rated, the ultimate goal is to make ListeningTime publicly available.

**Methods:**

First, members of a panel of elderly cancer survivors and patients (age ≥65 years) were approached to evaluate ListeningTime through a Web-based questionnaire. The usability and perceived usefulness were assessed. Second, ListeningTime was evaluated in real-life practice through a pilot study in 3 Dutch hospitals. In these hospitals, elderly patients with cancer and their oncological HCPs were approached to evaluate ListeningTime through a similar Web-based questionnaire, measuring the perceived usefulness. In addition, we examined log files and user statistics to get insight into how the program was used.

**Results:**

A total of 30 cancer survivors or patients from the patient panel, and 17 patients and 8 HCPs from the hospitals, evaluated ListeningTime. Overall, both panel members and hospital patients were positive about the ListeningTime website, audio-facility, and video fragments. Some patients suggested improvements with respect to the actors’ performances in the video fragments and believed that ListeningTime is mainly suitable for non experienced patients. HCPs were also positive about ListeningTime; they valued the video fragments for patients and the audio-facility for patients and themselves. However, providers did not relisten their own recorded encounters. Patients did use the audio-facility to relisten their encounters.

**Conclusions:**

ListeningTime was highly rated, both by patients and their oncological HCPs. As a result, the video fragments of ListeningTime are now made publicly available for elderly patients with cancer through the Dutch website “kanker.nl.”

## Introduction

Effective patient-provider communication is an important condition to deliver optimal care, and it supports patients in coping with their disease. The complex and emotionally loaded setting of oncology care challenges both health care providers (HCPs) and patients in reaching effective communication. Elderly patients with cancer find it difficult to communicate their informational needs or preferences, and, in general, their participation during interactions with HCPs is low [1,2]. In a recent study, 47% of elderly patients with cancer reported barriers in communicating with their oncological HCP, for example, not wanting to be bothersome, remembering topics to discuss only afterwards, and feeling nervous [3]. In addition, HCPs not always check whether or not patients understand the information, do not continuously explore what patients already know, and what information they still need [4,5]. In this vulnerable setting, elderly patients are additionally challenged by age-related deficiencies, like comorbidity, memory loss, hearing and vision problems, and having a smaller network [6,7]. These age-related deficiencies can hinder the interaction with HCPs and have an impact on the outcomes of the communication, as information recall [1]. They require sensitive communication of HCPs, taking patients’ needs into account.

These findings indicate the importance of supporting both HCPs and elderly patients with cancer in their communication. Preparing an encounter by watching modeling videos, that is, demonstrating different communication strategies of simulated patient-provider encounters, has been found to have positive effects on the quality of patient-provider interactions [8-10]. Relistening an audiorecording of one’s own clinical encounter is another intervention that has proven to support patients in various ways—by enhancing recall, improving decision making and the communication with family members, and reducing anxiety [11-13]. With the aim to overcome communication barriers by having elderly patients with cancer and their oncological HCPs (better) prepare the clinical encounter, we combined these 2 techniques and developed ListeningTime, a Web-based preparatory communication tool, based on needs assessment among elderly patients with cancer and their oncological HCPs [3,14]. A Web-based intervention was chosen, as the internet is a valuable source of information and support, also for elderly patients with cancer [15,16]. In the Netherlands, 88.3% of the elderly aged ≥65 years use the internet [17]. In addition, the content of Web-based interventions can be computer-tailored to patients’ needs and preferences; Web-based interventions are easily accessible and time-efficient and the cost of implementation is minimal once developed [18,19].

ListeningTime contains 2 video diaries, with each 12 short video fragments of simulated patient-HCP encounters. The video fragments demonstrate different communication strategies. At the end of every fragment, a simulation question is formulated (eg, “what would you do if...emotions get in your way/you do not understand what your doctor is talking about?”). Patients are asked to watch a set of 6 personally relevant video fragments, selected by an algorithm. HCPs are asked to watch one entire diary with 12 fragments. Patients and HCPs can furthermore relisten their audiorecorded encounter through the available audio-facility. Moreover, they can access the website anywhere, at any time, with a personal log-in. The participatory development process of ListeningTime is described in a previous publication [14].

A problem with many electronic health (eHealth) interventions is that they often remain unused after being developed. One of the reasons is that in daily practice, the intervention is not easy to use. The usability and perceived usefulness are preconditions for the actual use of websites like ListeningTime.

Therefore, this study aims to evaluate the usability, perceived usefulness, and actual use of ListeningTime, through the eyes of elderly patients with cancer and their oncological HCPs. The ultimate goal is, in case of high rating of ListeningTime, to make this Web-based communication tool publicly available.

## Methods

### Aim of ListeningTime

ListeningTime, a Web-based preparatory communication tool for elderly patients with cancer, was developed to help patients (better) prepare their encounters with oncological HCPs. In addition, the tool was designed to support HCPs in preparing their encounters with elderly patients. An overarching aim of the project was to develop ListeningTime in a participatory way to increase its uptake and use. The participatory development process of ListeningTime, including the content and techniques used, was extensively described in a previous publication [14]. In short, ListeningTime is a website, containing 2 video diaries of simulated patient-HCP encounters in which different communication strategies are demonstrated. Patients are asked to watch a selection of personally relevant video fragments, based on an algorithm. HCPs are asked to watch one entire diary. Furthermore, the website contains an audio-facility. Patients and HCPs can relisten their audiorecorded encounter through the facility. For this study, ListeningTime was evaluated in real-life clinical practice among both patients and providers.

### Design

A cross-sectional design was used to evaluate the usability, perceived usefulness, and actual use of ListeningTime, according to and by elderly cancer survivors and patients and their oncological HCPs. First, members of a patient panel were approached to evaluate ListeningTime through a Web-based questionnaire. Second, ListeningTime was evaluated in real-life clinical practice through a pilot study in Dutch hospitals, using a Web-based questionnaire and examining user statistics and log files.

### Ethics

This study was conducted according to the Dutch privacy legislation. According to the Dutch legislation, approval by a medical ethics committee was not required. Participation was voluntary, and participants gave their informed consent at the start of their participation.

### Recruitment

Elderly patients with cancer were approached through the Dutch patient panel “kanker.nl” (translated as “cancer.nl”); this patient panel consists of 169 cancer survivor or patients aged ≥65 years, of which 88 were invited to evaluate ListeningTime. In March 2016, they were invited to fill in a Web-based questionnaire to evaluate ListeningTime. They were asked to navigate through the website while answering the questions. Oncological HCPs (ie, oncologists and oncology nurses) from 3 Dutch hospitals were invited to partake in the pilot study to evaluate ListeningTime.

At the start, HCPs were asked to visit the website ListeningTime, create a personal log-in account, sign the digital informed consent, fill in a baseline questionnaire, and watch one of the 2 video diaries of simulated patient-HCP encounters containing 12 short video fragments (Textbox 1). After HCPs had watched the video diary (or diaries), they were asked to include patients for the pilot study.

From April to December 2016, HCPs approached eligible patients during their medical visits, and handed out a leaflet to patients asking to visit the website ListeningTime before their next visit. Patients were eligible if they were aged ≥ 65 years, diagnosed with cancer, had internet access, spoke and read Dutch, and were not in the palliative or terminal phase of the disease. Interested patients who visited the website were informed about the study, instructed on the website to create a personal log-in and sign a digital informed consent form and fill in a baseline questionnaire to get access to the selection of 6 personally relevant video fragments. The selection of 6 personal relevant video fragments and the order of the fragments varied per patient, based on the algorithm. The algorithm was based on the level of patients’ confidence in communication with the oncological HCP (through the Perceived Efficacy in Patient-Physician Interactions Questionnaire [20]), the importance of discussing several subjects (eg, quality of life, intimate issues as based on patients’ needs assessment [3]) and their sex (male or female).

**Figure 1:**
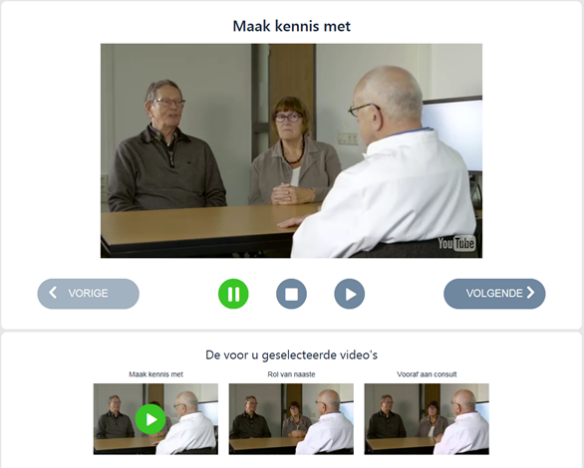
Screen captures of ListeningTime video fragments.

Fragments 1 and 2 were always offered as first 2 fragments (Textbox 1). The stories of patients in the 2 diaries differed (ie, one diary tells the story of a female patient with lymphoma, the other diary that of a male patient with prostate cancer) and also their participation level during the stimulated encounters differed (ie, one diary represents a more “active or assertive” patient, the other diary a more “passive” patient). Figure 1 shows a screenshot of the video fragments.

On the informed consent form, patients could opt for audiorecording of their next encounter with their oncological HCP. In case of consent, their HCP audiorecorded this next visit and uploaded the recording on ListeningTime; this enabled patients, their spouses, and HCPs to relisten their audiorecorded encounter, using their personal log-in.

Within 1 week after the (audiorecorded) visit to their HCP, patients were asked to evaluate ListeningTime through a Web-based questionnaire. At the end of the study, HCPs were also asked to evaluate ListeningTime through a Web-based questionnaire.

### Web-based Questionnaires

The Web-based questionnaire of the patient panel was used to assess the usability and perceived usefulness of ListeningTime; this questionnaire inquired about patients’ sociodemographic characteristics, their first impression of the website, textual parts of the website, log-in procedure, video fragments, audio-facility, and other remarks. The Web-based questionnaire in the pilot study was used to evaluate the perceived usefulness of ListeningTime, according to patients and oncological HCPs in hospital-based care. Furthermore, this questionnaire assessed the textual parts of the website, log-in procedure, video fragments, audio-facility, and other remarks.

**Overview of the topics of the video fragments.**

1. Introduction patient and companion
2. The role of the companion
3. Emotions
4. Choices about treatment options concerning the quality of life
5. Remembering information
6. Need for support
7. Prior to the encounter
8. Asking questions (about prognoses; where treatment takes place; wait-and-see policy; intimacy or sexuality; fear of dead)
9. Indicating your complaints or concerns
10. Asking all your questions
11. Complex information
12. Various information sources

### Usability

The usability of ListeningTime was measured with the System Usability Scale (SUS) [21]. The SUS includes 10 items about several facets of usability, for example, the complexity of the website and the ease of using it, scored on a 5-point Likert scale, ranging from 0 (strongly disagree) to 4 (strongly agree). SUS scores were calculated following the guidelines from the original publication [21]. As individual items of the SUS are not meaningful on their own, a total SUS score will be calculated. SUS scores range from 0 to 100; higher scores indicate higher usability. A previous study, evaluating nearly 10 years of SUS data collected, indicated that the SUS is a highly robust and versatile tool and also provides details on what constitutes an acceptable SUS score [22].

### Perceived Usefulness

The perceived usefulness of ListeningTime, that is, “the degree to which a person believes that using a particular system would enhance his or her job performance” [23], was measured using questions and statements. Similar questions and statements were used in previous studies [24,25]. Multimedia Appendix 1 describes the questions that were asked through the patient panel (18 questions), and questions and statements included in the pilot study (14 questions or statements for patients; 13 questions or statements for HCPs).

### Use

The actual use of ListeningTime by patients and HCPs in the pilot study was examined using user statistics and log files, that is, automatically generated files mapping the interactions between program and users; this allowed us to get insight into what extent patients and HCPs actually used the website, including the log-in frequency, playing video fragments, and using the audio-facility.

### Overall Rating of ListeningTime

We considered the rating of ListeningTime “high” in case ≥70% of cancer survivors or patients perceived ListeningTime as useful (in both the patient panel and pilot study), the usability was rated as “good” or higher [22], and 70% of the included patients actually used ListeningTime (ie, logged on, watched the video fragments) in the pilot study [26]. As the use of the audio-facility was optional, we considered the rating of the audio-facility “high” in case all patients who made use of it found it useful.

### Implementation Strategy

The ultimate goal was to implement ListeningTime, in case of high rating, as a publicly available, standalone intervention, that is, without the research context and without support of professionals. Therefore, we collaborated from the start of the project with several partners. This participatory development method was pursued to create awareness of the potential of ListeningTime and to prepare for a successful implementation (see for more details about the participatory development process of ListeningTime [14]). These partners included representatives from hospitals, the Nederlandse Federatie van Kankerpatiënten organisaties, the “Quality institute for oncological and palliative research and practice” (IKNL: Integraal Kankercentrum Nederland) and “kanker.nl” During the project, implementation of ListeningTime by one or several of these partners was discussed.

### Statistical Analyses

Descriptive statistics were used to analyze the results. Data analyses were performed in Stata version 14.

## Results

### Patient Panel

#### Study Sample

Of 88 members of the patient panel who were invited to evaluate ListeningTime, 30 members responded and filled in all questions. Respondents were on average aged 69 (range 65-78) years, 73% (22/30) were males, 43% (13/30) were highly educated (ie, higher professional education or university), and 83% (25/30) were married or had a registered partnership. In addition, 60% (18/30) were diagnosed with urological cancer (kidney, prostate, and bladder); 40% (12/30) indicated that they were currently being treated for cancer, and 33% (10/30) had completed treatment. Other respondents were awaiting treatment, following a wait-and-see policy or indicated that they completed treatment.

#### Usability of the Website

Patients had a mean SUS score of 73.2 (SD 18.5, range 30-100, n=30), which indicates good usability [21,22].

#### Perceived Usefulness of the Website

At first impression, 50% (15/30) of respondents found the website clear, 43% (13/30) found the website reliable, 37% (11/30) professional, 17% (5/30) inviting, and 13% (4/30) attractive. The website was not experienced as boring, busy, gloomy, or confusing.

Next, respondents evaluated the subpages of the website: “About ListeningTime” and “Patients.” Overall, 83% (25/30) of respondents could easily find the page “about ListeningTime,” 97% (29/30) found it clear to whom the website is intended, 93% (28/30) found it clear what the website has to offer, and 83% (25/30) stated that they did not miss any information about ListeningTime. Respondents who missed information (n=3) indicated that the website lacked information about a second opinion, how to inform more experienced patients, and which hospitals are cooperating with this research. The information about the participating hospitals was added to the website, and the topic of a second opinion was included in the script of a diary.

In addition, 80% (24/30) of respondents were able to easily find the page “patients.” Seven respondents explicitly mentioned that the page is clear, clean, well designed, and easy to search. Two respondents indicated that the amount of text could be less. Therefore, the amount of text on the website was reduced to a necessary minimum.

Respondents made the following, partly contrary remarks about the website:

a good website with many possibilities

I thought it was a bit boring and educational, I hope this will not stop people from using it

if possible, implementation via the website ‘kanker.nl’

video fragments were very weak

nice addition to the information from oncology

#### Perceived Usefulness of the Video Fragments

In this study, 70% (21/30) of respondents were able to watch the video fragments. The remaining respondents did not log-in to watch the video fragments (n=5), indicated to watch the video fragments another time (n=2), were abroad (n=1), or too emotional to watch the video fragments (n=1). Almost all respondents were satisfied with the selection and playing of the video fragments. They made the following comments about the video fragments: *easy; good; simply click; fine; without hesitation or interruption; sound was pleasant and clearly spoken; video’s played without problems.*

In addition, almost all were satisfied with the “simulation questions” (eg, “what would you do if...emotions get in your way/you do not understand what your doctor is talking about?”) at the end of every video fragment. Respondents stated the following about the simulation questions: *clear; fine; encourage thoughts; focus; encouragement to watch the video again; very personal questions; good questions but not complete*. Five respondents missed the question or did not watch the entire fragment.

Respondents found the video fragments easy to follow (20/21, 95%), clear (19/21, 90%), clearly spoken (18/21, 85%), good (17/21, 81%), realistic (16/21, 76%), credible (16/21, 76%), simple (15/21, 71%), reliable (15/21, 71%), complete (14/21, 67%), professional (12/21, 57%), and instructive (8/21, 38%). Among other things, they found the following things “good” about the video fragments: *recognizable; realistic; simplicity and clarity; dialogue; calm; well structured; HCP asks for and gives correct answers; effective; clear step by step method; good idea of how to communicate with the HCP and to bring certain aspects to their attention; powerful; very accommodating to the patient; answer to some questions.*

Respondents mentioned the following improvement points: *acting performance; more depth; identification with actors was not present (although maybe not necessary); more realistic situations (* eg, *bad news conversation).*

#### Perceived Usefulness of the Audio-Facility

Most respondents (21/30, 70%) were (very) enthusiastic about the possibility to audiorecord their conversation with the HCP and relisten this recording on the website. Respondents mentioned the following: *very commendable; excellent idea; awesome; fantastic; it would be very nice to take this opportunity; after a while you forget things or you do not know exactly what has being said, so this is a good thing*. Nine respondents were not interested in this because they already made their own recordings, brought a companion to the encounter, did not feel the need to record their encounter, found it a violation of the privacy of the HCP.

#### Perceived Usefulness of ListeningTime

Overall, 40% (12/30) of respondents would like to follow the entire program of ListeningTime, 20% (6/30) were considering it, and 40% (12/30) were not interested. In addition, 83% (25/30) found ListeningTime, or a similar program where patients see video fragments as an example of how certain topics can be discussed with their HCP, helpful for patients. Five patients did not agree and preferred a personal conversation with their HCP or found the video fragments to superficial because they already had a lot of (disease) experience.

This [ListeningTime] can help in processing

I think this is much clearer than reading information in a folder

You know what to ask for

It is a kind of training and sometimes a patients does not think of everything, especially when there is a lot of emotion

You can prepare your encounter with the video examples

### Pilot Study in Hospitals

#### Study Sample

A total of 17 patients and 8 oncological HCPs participated in this part of the study. Overall, 88% (15/17) of patients were treated for their disease, 1 patient had just undergone surgery, and 1 patient was in remission.

Two of the HCPs (one per hospital) included patients for the study. Seven of the HCPs completed the evaluation questionnaire after completing the communication training (ie, watching one entire diary of ListeningTime). Tables 1 and 2 present the characteristics of participants and health care providers, respectively.

#### Perceived Usefulness of the Website

Patients considered the website easy to use (17/17, 100%), clear (17/17, 100%), interesting (14/17, 82%), and well designed (15/17, 91%). All patients indicated to (probably) recommend the website to other patients. Moreover, 91% (15/17) of patients considered ListeningTime as useful for patients.

All HCPs found the website interesting, nicely designed, well organized, and easy to use. In addition, 43% (7/17) would recommend the website to colleagues, and 86% (15/17) of HCPs missed no information. One HCP indicated that written information on the website about what is important in communication could be added.

Close to reality

patient

You know what you can and may ask

patient

Remembering easier what the doctor has told

patient

**Table 1:** The characteristics of patients (n=17).

Characteristics		Value
Age in years, mean (range)		74 (66-89)
Male, n (%)		9 (53)
High educational level^a^, n (%)		6 (35)
**Household size, n (%)**		
	1	6 (35)
	2	11 (65)
**Diagnosis, n (%)**		
	Stomach, liver, or bowel cancer	9 (53)
	Breast cancer	6 (35)
	Gynecological cancer	1 (6)
	Unknown	1 (6)
**Attending clinical encounters, n (%)**		
	Always alone	2 (12)
	Sometimes alone	1 (6)
	Always with companion	14 (82)

^a^Higher professional education or university.

**Table 2:** The characteristics of health care providers (n=8).

Characteristics		Value
Age in years, mean (range)		42 (31-61)
Male, n (%)		1 (13)
Working experience in years, mean (range)		7 (1-17)
**Profession, n (%)**		
	Nurse	5 (63)
	Medical oncologists	2 (25)
	Doctors assistant	1 (12)

#### Perceived Usefulness of the Video Fragments

In this study, 10 patients watched the video fragments before their oncological encounter, 1 only after the encounter. Five patients watched the fragments again after the encounter. On average, patients watched the fragments 1.4 times (range 1-3). The video fragments were considered well designed (10/11, 91%), useful (10/11, 91%), interesting (9/11, 82%), realistic (9/11, 82%), and informative (8/11, 73%).

In addition, 86% (7/8) of HCPs found the video fragments nice, 50% (4/8) found them interesting, and 29% (2/8) found the fragments useful for themselves and realistic. HCPs indicated that the reactions of oncologists in the fragments were not always feasible in practice (eg, taking a pause in-between a conversation) or that they could not find out which learning moments there were for patients and were, therefore, curious about the evaluation by patients. Furthermore, 86% (7/8) of HCPs thought that a program such as ListeningTime could be helpful for patients; they indicated that the fragments are not useful for themselves, but may be for patients.

The video fragments are clear, but for more experienced patients not very much to the point.

patient

The fragments are too simple. Most patients are already familiar with the tips that were given in the fragments.

HCP

#### Perceived Usefulness of the Audio-Facility

Eight patients indicated that they audiorecorded their encounter and replayed it on the website. Three patients relistened alone, 3 with their spouse, and 2 relistened twice—once alone and once with their spouse. All patients considered relistening their encounter as useful for themselves and their spouse, and it all helped them to remember the conversation with their HCP.

As a patient, it is very useful to listen back to your encounter, good service.

All HCPs indicated that they did not relisten the audio recordings of their conversations with patients; this was confirmed by the user statistics. One HCP did not feel the need to relisten the audiorecordings and other HCPs did not find it useful to relisten all audio recordings. Nevertheless, HCPs were positive about the possibility of recording conversations. They indicated that the recording of the conversation is useful for themselves and patients and that it provides insight into their own communication skills.

#### Use of ListeningTime by Patients

The user statistics show that 17 patients logged on to the website, 5 times on average (range 1-17). Furthermore, 4 patients relistened the full audiorecording of their encounter, and other patients listened to a part of their audiorecorded encounter.

In addition, the user statistics show that 12 patients (12/17, 71%) fully watched ≥1 video fragments. On average, they viewed 9 fragments (range 1-20) *.* Of note, 4 of 12 patients viewed the 6 personally selected fragments, as intended. The introductory fragments about patients and the role of the companion were viewed by almost all patients (as intended part of the algorithm). Next, patients fully viewed the following fragments (≥1 times; in order of frequency)—choices about treatment options concerning the quality of life (n=12, diary 1: 6 patients, diary 2: 6 patients); emotions (n=11, diary 1: 5 patients; diary 2: 6 patients); remember information (n=11, diary 1: 5 patients; diary 2: 6 patients); and need for support (n=10, diary 1: 6 patients; diary 2: 4 patients). The following fragments were watched by <4 patients: prior to the encounter; asking questions (about prognoses, where treatment takes place, wait-and-see policy, intimacy and sexuality, and fear of death); indicate your complaints or concerns; asking all your questions; complex information and various information sources.

Three patients watched some of the video fragments. In particular, they looked at the fragments about “choices about treatment options concerning the quality of life” and “remembering information.”

#### Implementation

As mentioned before, the ultimate goal was to implement ListeningTime as a publicly available, standalone intervention, without the research context and he involvement of professionals. As of June 2017, the educational video fragments of ListeningTime are publicly available for all (elderly) patients with cancer through the Dutch website “kanker.nl.”

## Discussion

### Principal Findings and Comparison With Prior Work

ListeningTime is a useful and user-friendly communication tool for elderly patients with cancer. It helps patients to (better) prepare the clinical encounter with their oncological HCP and overcome communication barriers. Patients most valued the video fragments and the audio-facility to relisten their recorded consultations. They mentioned that ListeningTime supported their informational needs (eg, know what you can ask), emotional needs (eg, how to deal with emotions and ask for support), and their cognitive needs (eg, better remember what the doctor has told).

Patients often feel emotionally overwhelmed after diagnosis or during cancer treatment and have a need for emotional support. In addition, most patients with cancer report difficulties in understanding and fully processing the HCPs’ information [27,28]. ListeningTime seems to offer an opportunity to fulfill these needs.

Previous research found that combining audiovisual information with conversational style is the best way to present eHealth information about cancer treatment to (younger and older) adults [29]; this can explain patients’ high rating of ListeningTime as we used a combination of audiovisual information with conversational style in the video fragments. However, for more experienced patients, the video fragments seem less useful. Future research is necessary to get insight into which moment is or are (most) appropriate to use ListeningTime (eg, at the start of a disease trajectory).

Likewise, oncological HCPs were positive about ListeningTime as a supportive tool for patients. They valued the video fragments and the possibility to relisten the audiorecorded consultation. However, they also mentioned that the video fragments were too simple for patients. It is possible that HCPs overestimate their patients’ communication skills or that they included mainly experienced patients during the pilot study. Analyzing real-life, video- or audiorecordings of patient- provider encounters in this setting can provide insights into the communication process and role of both patients and providers. As only 8 patients audiorecorded their encounter during this study, it is not possible to draw conclusions. As mentioned before, HCPs were positive about the possibility to relisten the audiorecordings. However, they did not relisten their own recorded encounters; this could be attributed to several reasons, for example, owing to the lack of time or not feeling the need to relisten. For this study, the main aim was to support elderly patients with cancer in overcoming their communication barriers; ListeningTime seems to offer this opportunity. Although oncological HCPs participated in this study to support patients in their communication skills and, therefore, used ListeningTime, we did not offer a specific communication training for HCPs. The high use of ListeningTime by patients, however, can also be attributed to the involvement of HCPs in including patients and asking them to visit the website. Over recent years, many eHealth interventions have been developed. However, numerus eHealth interventions have not been evaluated, have reported attrition (like dropout and nonusage) and adoption problems (ie, poor uptake after implementation) [26,30,31]. By actively involving elderly patients with cancer and their providers in developing ListeningTime, the use and uptake of this intervention was expected to increase [32,33]. The evaluation of ListeningTime, indeed, showed that patients valued ListeningTime and, as a result, the video fragments became publicly available for all elderly patients with cancer. A previous study found similar results and concluded that actively involving patients with cancer in designing and evaluating a Web-based tool is feasible and appreciated [34]. For the design of the website, guidelines for targeting elderly patients online were followed, that is, avoiding large amounts of text by using “pull out” menus for more detailed information and larger font size [16]; this could have supported the use of ListeningTime.

Although ListeningTime was developed to support elderly patients with cancer especially, the tool might be very useful for younger patients as well. A recent study found no differences in website satisfaction between younger and older patients with cancer using a mode-tailored website [35]. Nevertheless, it should be tested if ListeningTime is also useful for younger patients with cancer. To this extent, it would be interesting to know how many (elderly and younger) patients (and their significant others) use the educational video fragments of ListeningTime since the implementation on the website “kanker.nl.”

In this study, we evaluated a Dutch Web-based communication tool. However, the results might be useful and relevant at the international level as well. As our results indicate, a tool as ListeningTime can be highly valuable to offer to elderly patients with cancer. It consists of multiple useful techniques, that is, a tailoring algorithm, modeling videos (including simulation questions), and an audio-facility [14], which can be useful for other countries and settings as well.

To the best of our knowledge, this is one of the first studies using a participatory process to develop a Web-based intervention, that is, with the help of elderly patients with cancer and their providers [30,34]. In addition, the educational video fragments of ListeningTime were implemented through the website “www.kanker.nl.” This success can be attributed to the participatory nature of the development process and the inclusion of partners from the start of the project. Unfortunately, it was not technically possible to include the tailoring algorithm and the audio-facility of ListeningTime on the website of “kanker.nl.” For further implementation of ListeningTime, the involvement of HCPs (or hospitals) might be necessary. Unfortunately, this was beyond the scope of this project.

For future research, it might be interesting to investigate the (combined) effect of the video fragments and audiorecordings on real-life communication between patients and HCPs; examine the effect of the simulation question at the end of each video fragment (eg, how do patients use or reflect on these questions, is it a crucial part of the video fragment, what is a good simulation question); explore other ways to provide patients with educational videos and audiorecordings of their clinical encounters; and how to implement interventions like ListeningTime in close collaboration with HCPs. A necessary first step before developing eHealth interventions is to investigate if the targeted patient population feels the need for the proposed eHealth intervention.

### Limitations

This study has some limitations. First, the results may be influenced by the relatively small study sample. However, this is an exploratory pilot study. Larger, controlled studies are necessary to replicate (or contradict) our findings. Second, it is possible that only interested patients participated. However, this is also the targeted group that will watch the video fragments on “kanker.nl.” Third, we aimed to include a wide range of elderly patients with cancer, with different (stages of) disease and different levels of participation (eg, active and passive). As patients volunteered to partake, it is possible that the results of this study represent the more “active” patients—those who feel confident in participating during medical encounters. In addition, we are not aware of the number of patients approached by providers in the hospital and the number nonresponders.

### Conclusions

ListeningTime was highly rated, both by elderly patients with cancer and their oncological HCPs. As a result, the video fragments of ListeningTime are publicly available for all (elderly) patients with cancer through the Dutch website “kanker.nl,” without the research context and the involvement of professionals.

## Appendix

Multimedia Appendix 1 Perceived usefulness questions and statements.

## Abbreviations

eHealth: electronic health
HCP: health care provider
SUS: System Usability Scale

## References

[1] Jansen J, van Weert Julia C M, de Groot Judith, van Dulmen Sandra, Heeren TJ, Bensing JM (2010). Emotional and informational patient cues: the impact of nurses' responses on recall. Patient Educ Couns.

[2] Posma ER, van Weert Julia C M, Jansen J, Bensing JM (2009). Older cancer patients' information and support needs surrounding treatment: An evaluation through the eyes of patients, relatives and professionals. BMC Nurs.

[3] Noordman J, Driesenaar JA, Henselmans I, Verboom J, Heijmans M, van Dulmen S (2017). Patient participation during oncological encounters: Barriers and need for supportive interventions experienced by elderly cancer patients. Patient Education and Counseling.

[4] Bensing JM, Deveugele M, Moretti F, Fletcher I, van Vliet Liesbeth, Van Bogaert Marjolein, Rimondini M (2011). How to make the medical consultation more successful from a patient's perspective? Tips for doctors and patients from lay people in the United Kingdom, Italy, Belgium and the Netherlands. Patient Educ Couns.

[5] Pieterse AH, Kunneman M, Engelhardt EG, Brouwer NJ, Kroep JR, Marijnen CAM, Stiggelbout AM, Smets EMA (2017). Oncologist, patient, and companion questions during pretreatment consultations about adjuvant cancer treatment: a shared decision-making perspective. Psychooncology.

[6] Brown SC, Park DC (2003). Theoretical models of cognitive aging and implications for translational research in medicine. Gerontologist.

[7] Williams SL, Haskard KB, DiMatteo MR (2007). The therapeutic effects of the physician-older patient relationship: effective communication with vulnerable older patients. Clin Interv Aging.

[8] Henselmans I, de Haes Hanneke C J M, Smets EMA (2013). Enhancing patient participation in oncology consultations: a best evidence synthesis of patient-targeted interventions. Psychooncology.

[9] Roter DL, Wexler R, Naragon P, Forrest B, Dees J, Almodovar A, Wood J (2012). The impact of patient and physician computer mediated communication skill training on reported communication and patient satisfaction. Patient Educ Couns.

[10] Krouse HJ (2001). Video modelling to educate patients. J Adv Nurs.

[11] Watson PWB, McKinstry B (2009). A systematic review of interventions to improve recall of medical advice in healthcare consultations. J R Soc Med.

[12] Hack TF, Ruether JD, Weir LM, Grenier D, Degner LF (2013). Promoting consultation recording practice in oncology: identification of critical implementation factors and determination of patient benefit. Psychooncology.

[13] Tsulukidze M, Durand M, Barr PJ, Mead T, Elwyn G (2014). Providing recording of clinical consultation to patients - a highly valued but underutilized intervention: a scoping review. Patient Educ Couns.

[14] Noordman J, Driesenaar J, van Bruinessen IR, van Dulmen S (2017). Internet Interventions.

[15] Bolle S, van Weert Julia C M, Daams JG, Loos EF, de Haes Hanneke C J M, Smets EMA (2015). Online Health Information Tool Effectiveness for Older Patients: A Systematic Review of the Literature. J Health Commun.

[16] Bolle S, Romijn G, Smets EMA, Loos EF, Kunneman M, van Weert Julia C M (2016). Older Cancer Patients' User Experiences With Web-Based Health Information Tools: A Think-Aloud Study. J Med Internet Res.

[17] CBS, Statline.

[18] Cegala DJ (2006). Emerging trends and future directions in patient communication skills training. Health Commun.

[19] Noar SM, Black HG, Pierce LB (2009). Efficacy of computer technology-based HIV prevention interventions: a meta-analysis. AIDS.

[20] Zandbelt LC, Smets EMA, Oort FJ, Godfried MH, de Haes HCJM (2006). Determinants of physicians' patient-centred behaviour in the medical specialist encounter. Soc Sci Med.

[21] Brooke J UK.

[22] Bangor A, Kortum P An empirical evaluation of the system usability scale. Int J Hum Comput Interact 2008;24(6):574-594.

[23] Davis FD (1989). Perceived Usefulness, Perceived Ease of Use, and User Acceptance of Information Technology. MIS Quarterly.

[24] Butow P, Devine R, Boyer M, Pendlebury S, Jackson M, Tattersall MHN (2004). Cancer consultation preparation package: changing patients but not physicians is not enough. J Clin Oncol.

[25] Albada A, Ausems MGEM, Otten R, Bensing JM, van Dulmen Sandra (2011). Use and evaluation of an individually tailored website for counselees prior to breast cancer genetic counseling. J Cancer Educ.

[26] Eysenbach G (2005). The law of attrition. J Med Internet Res.

[27] Rousseau SJ, Humiston SG, Yosha A, Winters PC, Loader S, Luong V, Schwartzbauer B, Fiscella K (2014). Patient navigation moderates emotion and information demands of cancer treatment: a qualitative analysis. Support Care Cancer.

[28] Mazor KM, Beard RL, Alexander GL, Arora NK, Firneno C, Gaglio B, Greene SM, Lemay CA, Robinson BE, Roblin DW, Walsh K, Street RL, Gallagher TH (2013). Patients' and family members' views on patient-centered communication during cancer care. Psychooncology.

[29] Bol N, van Weert Julia C M, de Haes Hanneke C J M, Loos EF, Smets EMA (2015). The effect of modality and narration style on recall of online health information: results from a Web-based experiment. J Med Internet Res.

[30] de Beurs D, van Bruinessen I, Noordman J, Friele R, van Dulmen S (2017). Active Involvement of End Users When Developing Web-Based Mental Health Interventions. Front. Psychiatry.

[31] van Gemert-Pijnen Julia E W C, Nijland N, van Limburg Maarten, Ossebaard HC, Kelders SM, Eysenbach G, Seydel ER (2011). A holistic framework to improve the uptake and impact of eHealth technologies. J Med Internet Res.

[32] Winterling J, Wiklander M, Obol CM, Lampic C, Eriksson LE, Pelters B, Wettergren L (2016). Development of a Self-Help Web-Based Intervention Targeting Young Cancer Patients With Sexual Problems and Fertility Distress in Collaboration With Patient Research Partners. JMIR Res Protoc.

[33] Omeni E, Barnes M, MacDonald D, Crawford M, Rose D (2014). Service user involvement: impact and participation: a survey of service user and staff perspectives. BMC Health Serv Res.

[34] van Bruinessen Inge Renske, van Weel-Baumgarten Evelyn M, Snippe HW, Gouw H, Zijlstra JM, van Dulmen Sandra (2014). Active patient participation in the development of an online intervention. JMIR Res Protoc.

[35] Nguyen MH, Smets EMA, Bol N, Loos EF, Van Weert Julia C M (2018). How Tailoring the Mode of Information Presentation Influences Younger and Older Adults' Satisfaction with Health Websites. J Health Commun.

