# Supplementary material for: Evaluation and Implementation of ListeningTime: A Web-Based Preparatory Communication Tool for Elderly Patients With Cancer and Their Health Care Providers
Source: JMIR Cancer. 2019 Jan 30;5(1):e11556. doi: 10.2196/11556 (PMC6372931; doi:10.2196/11556)
Supplement: Multimedia Appendix 1 [file cancer_v5i1e11556_app1.pdf]

Multimedia Appendix 1

Perceived usefulness questions and statements

The following questions were asked via the patient panel:

1. What is your first impression of the website? (please choose max. 3 words or give your own description: clear, professional, busy, attractive, boring, confusing, reliable, gloomy, inviting/ give your own description)

2. Were the subpages (‘about ListeningTime’, ‘patients’) easy to find? (yes/no)

3. Is it clear for who the website is intended? (no, not clear at all; no, not that clear; yes, pretty clear; yes very clear)

4. Is it clear what the website offers? (no, not clear at all; no, not that clear; yes, pretty clear; yes very clear)

5. Do you miss information? (no; yes, namely…)

6. Do you have remarks about this subpage (‘about ListeningTime’ respectively ‘patients’; open question)?

7. Did you succeed on logging in? (No, I could not login because…; Yes, but there were problems with logging in, namely…;Yes, the login went without problems)

8. Which video fragments did you watch? (open question)

9. How did you select the video fragments? (open question, with examples)

10. How did the videos play? (open question, with examples)

11. What did you think about the questions that appeared at the end of the video-fragments? (open question)

12. What did you think about the video fragments? (clear/unclear; wrong/good; complete/incomplete; amateurish/professional; easy/difficult; realistic/unrealistic; difficult to follow/easy to follow; instructive /not instructive; reliable/not reliable; difficult to understand/clearly spoken; not credible/credible)

13.What is good about the video fragments? (open question)

14. What would you like to see different concerning the video fragments? (open question)

15. What do you think of the possibility to audio record conversations with your oncology HCP and listen back to these recordings through the website? (open question)

16. Would you like to follow the entire program of ListeningTime if that would be possible? (No, because….;Maybe, because; Yes, because)

17. Do you think that ListeningTime, or a similar program where patients see video fragments as an example of how certain topics can be discussed with their HCP, can be helpful for patients? (No, because….; Yes, because).

18. Do you have other remarks about the website, videos and/or this questionnaire? (open question)

The questions and statements that were included in the pilot study for patients were:

1. Did you view the website/ video fragments prior to your consultation with your HCP? (No; Yes)

2. Why did you not view the website/ the video fragments prior to your conversation with your HCP? (I forgot; I did not have the time to visit the website/watch the video fragments; I did not want to view the website/video fragments; I could not open the website/video fragments due to technical problems; I was too ill/tired; other reason, namely…)

3.Did you view the website and video fragments (again) after your conversation with your HCP? (No; yes)

4. How many times did you view the website and the video fragments in total? (1 time; 2 times; 3-4 times; more than 4 times)

5. Do you want to indicate to what extent you disagree or agree with the following statements about the website? The website is: a. interesting; b. well designed; c. easy to use; d. slow; e. clear (totally disagree, disagree, agree, totally agree)

6. Do you want to indicate to what extent you disagree or agree with the following statements about the video fragments? The video fragments are: a. interesting; b. well designed; c. useful ; d. slow; e. realistic; f. informative (totally disagree, disagree, agree, totally agree)

7. Do you have other remarks about the website and/or the video fragments? (open question)

8. Did you miss information on the website? (No; Yes, namely…)

9. Would you recommend the website to others? (No; Maybe; Yes)

10. Do you think that ListeningTime, or a similar program where patients see video fragments as an example of how certain topics can be discussed with their HCP, can be helpful for patients? (No, because….; Yes, because).

11. Did you listen back to your audio-recorded consultation on your personal webpage? (No; Yes, I listened to the recording on my own; Yes, I listened to the recording together with a spouse; Yes, I listened to the recording alone and together with a spouse)

12. Why did you not listen back to your audio-recorded consultation? (I forget; I did not have time to listen back to my recorded consultation; I do not want to listen back to my recorded consultation; I could not listen back to my recorded consultation due to technical problems; I was too ill/too tired; other reason, namely…)

13. Do you want to indicate to what extent you disagree or agree with the following statements about the audio-recording of your conversation? The audio-recording is: a. useful for myself; b. useful for my family and acquaintences; c. unnecessary; d. helps me to remember the conversation (totally disagree, disagree, agree, totally agree).

14. Do you have other remarks about the audio-recording of your conversation?

The questions and statements that were included in the pilot study for oncological HCPs were:

1. Did you follow the entire program of ListeningTime (i.e. view all 12 video-fragments of 1 diary)? (No; Yes)

2.Why did you not view all the video fragments? (I did not have the time to view all video fragments; I did not want to view all video fragments; I only viewed the video fragments that I found relevant; I could not view the video fragments due to technical problems; other reason, namely..)

3. How many times did you view the video fragments in total? (1 time; 2 times; 3-4 times; more than 4 times)

4. Could you indicate to what extent you disagree or agree with the following statements about the website? The website is: a. interesting; b. well designed; c. easy to use; d. slow; e. clear (totally disagree, disagree, agree, totally agree)

5. Could you indicate to what extent you disagree or agree with the following statements about the video-fragments? The video-fragments are: a. interesting; b. well designed; c. useful ; d. slow; e. realistic; f. informative (totally disagree, disagree, agree, totally agree)

6. Do you have other remarks about the website and/or the video fragments? (open question)

7. Did you miss information on the website? (No; Yes, namely…)

8. Would you recommend the website to other HCPs? (No; Maybe; Yes)

9. Do you think that ListeningTime, or a similar program where patients see video fragments as an example of how certain topics can be discussed with their HCP, can be helpful for patients? (No, because….; Yes, because)

10. Did you listen back to one or more of the audio-recorded consultation on your personal webpage? (No, I did not listen back to a recording; Yes, I listened to one recording; Yes, I listened to multiple recordings; Yes, I listened to all the recordings)

11. Why did you not listen back to (all) the audio-recorded consultation(s)? (I forget; I did not have time to listen back to my recorded consultation; I do not want to listen back to my recorded consultation; I did not find it useful to listen to all the recorded consultations; I could not listen back to my recorded consultation due to technical problems; other reason, namely…)

12. Do you want to indicate to what extent you disagree or agree with the following statements about the audio-recording of your conversation? The audio-recording is: a. useful for myself; b. useful for the patient; c. unnecessary; d. provides insight into my communication skills (totally disagree, disagree, agree, totally agree).

13. Do you have other remarks about the audio-recording of your conversation?
